# Spatial analysis of human and livestock anthrax in Dien Bien province, Vietnam (2010–2019) and the significance of anthrax vaccination in livestock

**DOI:** 10.1371/journal.pntd.0010942

**Published:** 2022-12-20

**Authors:** Luong Minh Tan, Doan Ngoc Hung, Do Thai My, Morgan A. Walker, Hoang Thi Thu Ha, Pham Quang Thai, Tran Thi Mai Hung, Jason K. Blackburn

**Affiliations:** 1 Spatial Epidemiology and Ecology Research Laboratory (SEER Lab), Department of Geography, University of Florida, Gainesville, Florida, United States of America; 2 Emerging Pathogens Institute, University of Florida, Gainesville, Florida, United States of America; 3 National Institute of Hygiene and Epidemiology, Hanoi, Vietnam; 4 Provincial Center for Disease Control, Dien Bien Phu City, Dien Bien, Vietnam; 5 Provincial Sub-Department of Animal Health, Dien Bien Phu City, Dien Bien, Vietnam; 6 School of Preventive medicine and public health, Hanoi Medical University, Hanoi, Vietnam; University of Kelaniya Faculty of Medicine, SRI LANKA

## Abstract

Anthrax is a serious zoonosis caused by *Bacillus anthracis*, which primarily affects wild herbivorous animals with spillover into humans. The disease occurs nearly worldwide but is poorly reported in Southeast Asian countries. In Vietnam, anthrax is underreported, and little is known about its temporal and spatial distributions. This paper examines the spatio-temporal distribution and epidemiological characteristics of human and livestock anthrax from Dien Bien province, Vietnam from 2010 to 2019. We also aim to define the role of livestock vaccination in reducing human cases. Historical anthrax data were collected by local human and animal health sectors in the province. Spatial rate smoothing and spatial clustering analysis, using Local Moran’s I in GeoDa and space-time scan statistic in SaTScan, were employed to address these objectives. We found temporal and spatial overlap of anthrax incidence in humans and livestock with hotspots of human anthrax in the east. We identified three significant space-time clusters of human anthrax persisting from 2010 to 2014 in the east and southeast, each with high relative risk. Most of the human cases were male (69%), aged 15–59 years (80%), involved in processing, slaughtering, or eating meat of sick or dead livestock (96.9%) but environmental and unknown exposure were reported. Animal reports were limited compared to humans and at coarser spatial scale, but in areas with human case clusters. In years when livestock vaccination was high (>~25%), human incidence was reduced, with the opposite effect when vaccine rates dropped. This indicates livestock vaccination campaigns reduce anthrax burden in both humans and livestock in Vietnam, though livestock surveillance needs immediate improvement. These findings suggest further investigation and measures to strengthen the surveillance of human and animal anthrax for other provinces of Vietnam, as well as in other countries with similar disease context.

## Author summary

Anthrax is a bacterial disease, cause by *Bacillus anthracis*, that can transmit from herbivorous animals to humans through close contact, eating meat of infected animals, or through environmental contamination. Anthrax can be a severe disease requiring early diagnosis and treatment to avoid fatal outcomes, disability, or financial burden to patients and their families. The disease also affects human well-being when livestock is their livelihood and an important part in agricultural practice. Anthrax is preventable by vaccine but effective strategy for vaccine distribution should be built on the characteristics of the disease in both human communities and the local animal populations. This study provides evidence on the space-time distribution and other characteristics of anthrax applying a One Health approach and geographical information analysis techniques. We show that hotspots of human disease correspond to areas of animal case reporting. We also show human incidence is reduced with livestock vaccination rates are relatively high. The study results are useful for researchers to foster investigation on other aspects of the disease and control efforts. It also provides scientific evidence for advocacy and communication on the importance of anthrax vaccination and strategic planning on vaccine production and distribution in Vietnam and other countries with similar context.

## Introduction

Anthrax is an important zoonosis caused by *Bacillus anthracis*, a spore-forming, gram-positive bacterium, which primarily affects wild herbivores and domestic livestock [1], with spillover to humans most often associated with handling or consumption of contaminated animal productions [2–4]. Human anthrax is classified by route of infection including cutaneous (infection through the skin and lesion formation), gastrointestinal (consuming infected meat), inhalational anthrax (inhaling bacterial spores), and injectional anthrax (injecting *Bacillus anthracis* spore-contaminated heroin) [1,5]. The disease occurs nearly worldwide with human cases concentrated in countries where the livestock vaccine is not available or difficult to distribute at an adequate level to reduce disease burden [1,6]. Globally, sustained anthrax is reported across part of North America (the western USA and parts of Canada and Mexico), West and Sub-Saharan Africa, Europe, Central Asia, China and Southeast Asia [6].

In Asia, current evidence suggests anthrax is widespread throughout the continent, with more reported outbreaks from China, India, Iran, Mongolia, and countries of the former Soviet Union such as Kazakhstan, Uzbekistan [6–9]. In China, approximately 2000 human cases per year with a temporally decreasing trend were reported. The incidence and case fatality rate (CFR) were not proportional. While incidence reduced, a higher number of deaths and higher CFR were reported in late 1980s in China. The spatial distribution of anthrax outbreaks in China indicate anthrax is widespread in the northeast, central, southwest, and south regions of the country. Among those, the districts located in southern China share a long border with Vietnam [8]. Anthrax occurrence and distribution are poorly reported and understood in the Indochina region though there is some evidence of anthrax in humans and animals in Vietnam, Laos, and Cambodia [10–12].

In Vietnam, anthrax is nationally reportable, and disease control is reported and managed at the provincial level. Human anthrax cases are most often reported in the northern mountainous provinces such as in Dien Bien, Ha Giang, Lai Chau, Son La, Lao Cai and Cao Bang [13] where livestock grazing and trading at the border with China are common. The number of human cases ranged between 12 and 201 cases per year in the period of 2000–2014, and most of those were cutaneous anthrax [10]. Among those provinces, Dien Bien province has a northern border with the Yunnan province of China which witnessed a rebound of human anthrax cases despite the decreasing trend of other Chinese provinces during the same time period [7]. Dien Bien also shares a western and southwestern border with Lao People’s Democratic Republic (Lao PDR) [14]. The population of Dien Bien is diverse with 19 ethnic groups and various cultural practices that might influence farming practice and health seeking behaviors. It is one of the least developed provinces in Vietnam with 44.8% of the population living below the poverty line in 2016. Livestock production is one of the major sources of income for the province [15]. Though the disease is recurrent, data on human, livestock, or wildlife anthrax, or underlying factors driving disease recurrence are poorly understood in Vietnam, particularly in the northern region of the country, including Dien Bien province.

Vietnam has a national set of measures implemented in response to anthrax in Vietnam. For example, anthrax is listed in class B of infectious diseases with high possibility of transmission and fatality in the Law of Infectious Disease Prevention and Control number 03/2007/QH12 in 2007. However, anthrax has only been nationally reportable since 2015 following the development and publication of circular 54/2015/TT-BYT by the Vietnamese Ministry of Health (MOH). Under the policy, certain infectious diseases, including anthrax, must be reported into the national surveillance system within 24 hours of initial notification. Another inter-ministerial circular, number 16/2013/TTLT-BYT-BNN&PTNT (Circular 16), was issued in 2013 between MOH and Ministry of Agriculture and Rural Development (MARD), creating a legal framework for collaboration between the two sectors from local to central levels in efforts to prevent and control zoonoses, including anthrax. However, work is needed to fully implement regulations at provincial and lower administrative units (district, commune) [16].

In this paper, we evaluate the anthrax situation in the Dien Bien Province from 2010 to 2019 using historical data and reports of provincial human health and animal health sectors with the following objectives: 1) describe the spatio-temporal patterns and epidemiological characteristics of anthrax in humans and livestock across the province; and 2) compare vaccine coverage in livestock to anthrax reports in human and livestock. Spatial smoothing and spatial/space-time clustering analyses are employed to address these objectives.

## Methods

### Ethics statement

This study underwent ethical review by the Institutional Review Board in Bio-medical Research of the National Institute of Hygiene and Epidemiology, Vietnam (IRB-VN01057/IORG 0008555; Project IRB certificate number NIHE IRB-03/2020) and the University of Florida (IRB202003189).

### Study area

**Fig 1:**
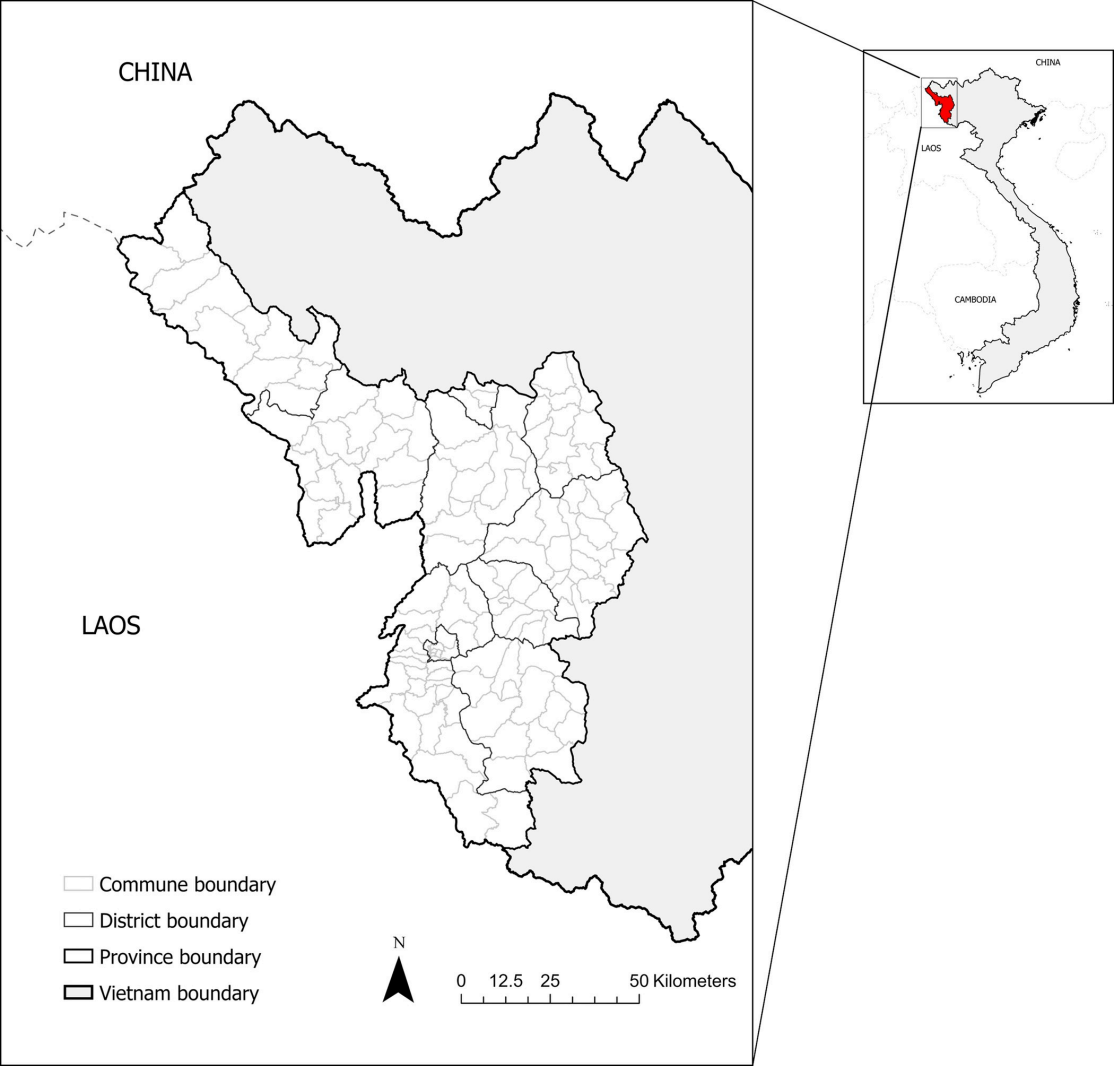
Districts and communes within Dien Bien province in northwestern Vietnam (produced in ArcGIS Pro using political boundary shapefiles from https://geodata.ucdavis.edu/gadm/gadm4.1/shp/gadm41_VNM_shp.zip).

Dien Bien is a remote mountainous province in the north of Vietnam, which has an east/southeast border with Son La province, northeast border with Lai Chau province, northern border with China, and west/southwestern border with Lao PDR (Fig 1) [14]. The population of Dien Bien is nearly 600,000 and comprised of 19 ethnic groups with diverse cultural practices. Dien Bien Phu City, the provincial capital, is ~500 kilometers from Hanoi, the national capital, and requires approximately 6–8 hours travel time by ground transportation. The same time is required for traveling from the province’s center to its remote districts (~200km), illustrating the difficulty of transportation within the province. Dien Bien is divided into 10 districts and 130 communes (sub-district). 116 of those communes are in rural and forestry areas (116/130, 89%) [14]. The three-level administration of Dien Bien province is illustrated in S1 Fig.

### Data collection and management

Crude numbers of human and livestock anthrax cases, and doses of livestock anthrax vaccine administered between 2010 and 2019 were collected by the Dien Bien Provincial Center for Disease Control (PCDC) and Sub-Department of Animal Health (Sub-DAH). Data were compiled into a database using communes (sub-district polygons) as the unit of spatial analysis for human anthrax and district level for animal anthrax. For this study, all spatial analyses were performed with political boundary data downloaded from GADM data version 3.6 *(www.gadm.org)* and all maps were prepared using ArcGIS Pro (ESRI, Redlands, California, USA). Here, the anthrax case definition was any case with clinical signs (humans and animals) and symptoms (humans) of anthrax. Human cases were considered for any of the four types of human anthrax including cutaneous, gastrointestinal, respiratory, and injectional anthrax with or without laboratory confirmation (S1 Text;). For this study space-time patterns were evaluated from 2010 to 2018. Human case data from 2019 were used to examine livestock vaccination success in reducing human disease.

### Population estimation for humans and livestock

For this study, human population data were estimated for each commune from gridded population counts retrieved from the WorldPop database using unconstrained individual countries UN adjusted population (*www*.*worldpop*.*org*) for each year from 2010 to 2019; the grid cells have resolution of ~100m x 100m at the equator [17,18]. The zonal statistics routine in ArcGIS Pro (ESRI, Redlands, California, USA) was used to estimate human population data for each commune polygon for each year. The zonal statistics application is described elsewhere [19]. Results for population estimates were plotted to compare with population estimations based on national censuses in 2009 and 2019 (S2 Fig), with an annual growth rate of 2% for Dien Bien province, and adjusted for 0.2% under-estimation rate reported nationwide [20]. S2A Fig shows that population estimated by Zonal statistics is about 1–3% higher than estimated population based on the two censuses and growth rates. We considered this acceptable as the error of national censuses in different countries ranged between 1.5–4.5% [20].

Livestock population, referred to as buffalo and cattle here, was calculated at the district level using the zonal statistics routine and raster data for global cattle distribution and global buffalo distribution in 2010 provided by Harvard Dataverse [21,22]. The grid cells have a resolution of 5 arc minutes or ~10 km x 10 km at the equator. Since the data were available for 2010, estimates for annual population were calculated based on growth rates of buffalo and cattle separately. According to gray literature from Dien Bien province, the annual growth rates of buffalo and cattle in 2010 were 4.45% and 11.8%, respectively [23]. We multiplied the buffalo and cattle in 2010 at district level by the growth rates to estimate the livestock population in 2011, and so on for each year from 2012 to 2018 with the assumption that the distribution of livestock among the districts varied slightly throughout the study period. To assess the accuracy of livestock population estimates, we compared the estimated total livestock at provincial level with total livestock provided by Dien Bien Sub-DAH. The results are presented in S2B Fig, which shows the underestimated livestock population between 2010–2014, and fairly accurate estimates in later years. The underestimates were considered in the data analysis and the interpretation of results.

### Cumulative incidence calculation and rate estimate smoothing

Cumulative incidence (CI) of human or animal anthrax was calculated by total cases (in humans or animals) per three years divided by the median human or animal population of each interval (2011, 2014, and 2017), then multiplied by 10,000. This resulted in three-year intervals of 2010–2012, 2013–2015, and 2016–2018. The CI represents the number of human or animal anthrax cases per 10,000 population in each time interval. Data from 2019 were used for other analyses but excluded from three-year interval calculations.

Rate smoothing was performed for human anthrax to stabilize the variability of crude CI caused by variation in numerators (number of cases) and denominators (population at risk) on later spatial autocorrelation analyses [24]. Given that anthrax is a rare disease, it varies largely among the communes, with many communes reporting zero cases while some reported high case numbers. Smoothing was conducted in GeoDa (version 1.20) with two methods including Empirical Bayes Smoothing (EBS) and Spatial Bayes Smoothing (SBS). The smoothing techniques are described elsewhere [24]. Briefly, both approaches aim to reduce rate variability by adjusting the estimates toward either a global mean (EBS) or local mean defined by a weights matrix (SBS); in both cases, the greatest adjustment is in polygons with low populations. Here we used a 1^st^ order queen contiguity matrix to define neighbors for the SBS, meaning any neighboring sharing a boundary line or vertex with the commune of interest would be considered a neighbor. The smoothed CI estimates from both methods were plotted using *par()* function in R-Studio to illustrate the change after smoothing [25]. S3 Fig shows that for human anthrax, SBS of CI collapsed outliers, the standard deviation, and the mean more than EBS of CI. Therefore, SBS CI was used for further spatial analysis. S Fig shows the choropleth maps of crude and SBS CI.

### Spatial autocorrelation analysis

Spatial autocorrelation analysis was conducted using SBS CI for human anthrax for each three-year interval in GeoDa version 1.20 [24]. Local Moran’s I, a local statistic derived from global Moran’s I by using Local Indicators of Spatial Association (LISA), was employed to identify spatial clusters of anthrax at the commune-level across the communes. The local Moran’s I is written following [26] as:

$$I_{i}=Z_{i}\sum W_{ij}Z_{j}$$

where *I*_*i*_ is the statistic for a commune *i*, Z_i_ is the difference between the anthrax risk at *i* and the mean anthrax rate for all of Dien Bien for that the three-year interval, *Z*_*j*_ is the difference between anthrax risk at commune *j* and the mean for Dien Bien. *W*_*ij*_ is the weights matrix. In this study, the 1^st^ order queen contiguity was employed. Here, *W*_*ij*_ equals 1/n if a commune shares a boundary or vertex and 0 if not. The statistic identifies spatial clusters of like values surrounded by like values, high-high for areas of clustered human anthrax or low-low where CI rates are lower than expected. The LISA also identifies spatial outliers, where values in *i* may be high surrounded by low values (high-low clusters), or the opposite, where low values in *i* are surrounded by high values (low-high). Significance of the statistical test was determined with pseudo p-value generated using 999 permutations against a null of complete spatial randomness [27]. Significant clusters were determined at p-value ≤0.05, which were mapped for each three-year interval in ArcGIS Pro.

### Space-time cluster analysis by SaTScan statistic

We were also interested in detecting space-time clusters for the full time period 2010 to 2018. Space-time cluster analysis was performed using SaTScan version 9.6 [28]. SaTScan uses a series of search circles of varying diameter with maximum diameter defined by a proportion of the population at risk (in Poisson model) to detect spatial clusters across study area and a series of cylinders varying heights to detect temporal clusters throughout the study period [27]. In this study, Poisson space-time scan statistic was conducted, with year as the time step (2010–2018). We evaluated three maximum diameter of search circles at 15%, 25% and 50% of population at risk. In each experiment, we set no geographical overlap among clusters, and the longitude and latitude of the centroid for each commune as location. The longitude and latitude were extracted with the Geometry tools in ArcGIS Pro. SaTScan clusters were determined at p≤0.05 level of significance. Relative risk (RR) was calculated based on the observed and expected value and the likelihood function of assumed distribution. Results were aggregated to the communes for mapping and dates of cluster persistence identified.

### Epidemiological description of anthrax in Dien Bien 2010–2018

Crude number of anthrax cases in human and livestock in each district of Dien Bien and crude annual incidence at the provincial level were plotted to show the temporal trend of the disease in both groups from 2010 to 2018. Crude cumulative incidences in each three-year period of both human and livestock anthrax were aggregated into polygons representing 130 communes of Dien Bien and choropleth mapped in ArcGIS Pro. Aside from the primary dataset between 2010 and 2018, we also had data on age, gender, and the source of infection for a subset of patients reported between 2012 and 2018.

### Incidence rates of anthrax in humans and livestock compared to annual livestock vaccine coverage

Total number of anthrax vaccine doses administered in livestock in Dien Bien province each year was provided by Dien Bien Sub-DAH. The vaccine was recommended with one dose per year for livestock in an endemic region [29]. Therefore, the annual vaccine coverage was calculated by dividing the total number of administered vaccine doses to estimated livestock population of corresponding year. Furthermore, biannual change in the incidence was also calculated as follows:

$$Biannual\;incidence\;change=\frac{y_{\left(i\right)}-y_{\left(i-1\right)}}{y_{\left(i-1\right)}}$$

Where y_(i)_ is the incidence of the calculated year; y_(i-1)_ is the incidence of the year prior to calculated year.

Human and livestock anthrax incidence (per 10,000) were plotted together with annual vaccine coverage (percent) or biannual incidence change (percent) in line graph to illustrate the association between vaccine coverage and disease trend in both subjects.

## Results

### Human and livestock population growth between 2010–2018

Both humans and livestock were more populated in southern districts of the province. The increase in human population was seen throughout the province but more intense in southern districts (S5 Fig.

### Spatial clusters and outliers of human anthrax between 2010–2018

**Fig 2:**
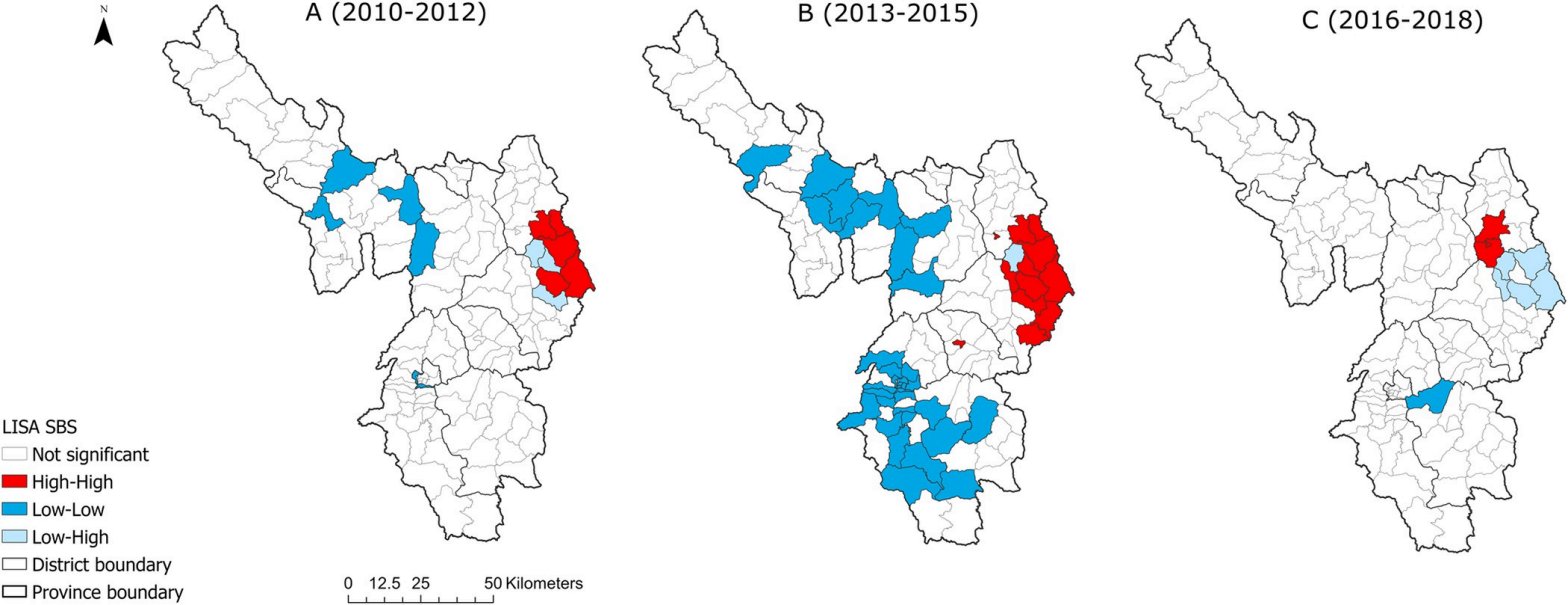
Local Moran’s I defined spatial clusters of human anthrax incidence (per 10,000) per commune for 3-year intervals, Dien Bien province, Vietnam. Incidence values were smoothed using the Spatial Bayes routine in GeoDa (detailed Local Moran’s I statistics in S6 Fig. Maps produced in ArcGIS Pro using political boundary shapefiles from https://geodata.ucdavis.edu/gadm/gadm4.1/shp/gadm41_VNM_shp.zip).

Local Moran’s I defined spatial clusters of smoothed human anthrax cumulative incidence per three-year interval are presented in Fig 2. Clusters and spatial outliers of human anthrax were identified in each of the three intervals. Overall, there was a hotspot of human anthrax in the eastern part of Dien Bien province throughout the 9-year period. Between 2010–2012, there were significant hotspots of human anthrax in the eastern parts (location of high value surrounded by other locations of high value) and cold spots (location of low value surrounded by other locations of low value) in the north and southwest of the province. There were low-high outliers of anthrax in eastern communes. In the second period of 2013–2015, the hotspot was still in the east but expanded in size. Additionally, two new hotspots constituted by two separate communes were seen in this period. The cold spots of anthrax also expanded to a wider area in the north and south of Dien Bien province. There was an outlier of low-high values in the east. In the last period, the hotspot remained in the east but contained fewer communes. The communes with high values in former periods became outliers of low values in the last period.

### Space-time clusters of human anthrax between 2010–2018

**Fig 3:**
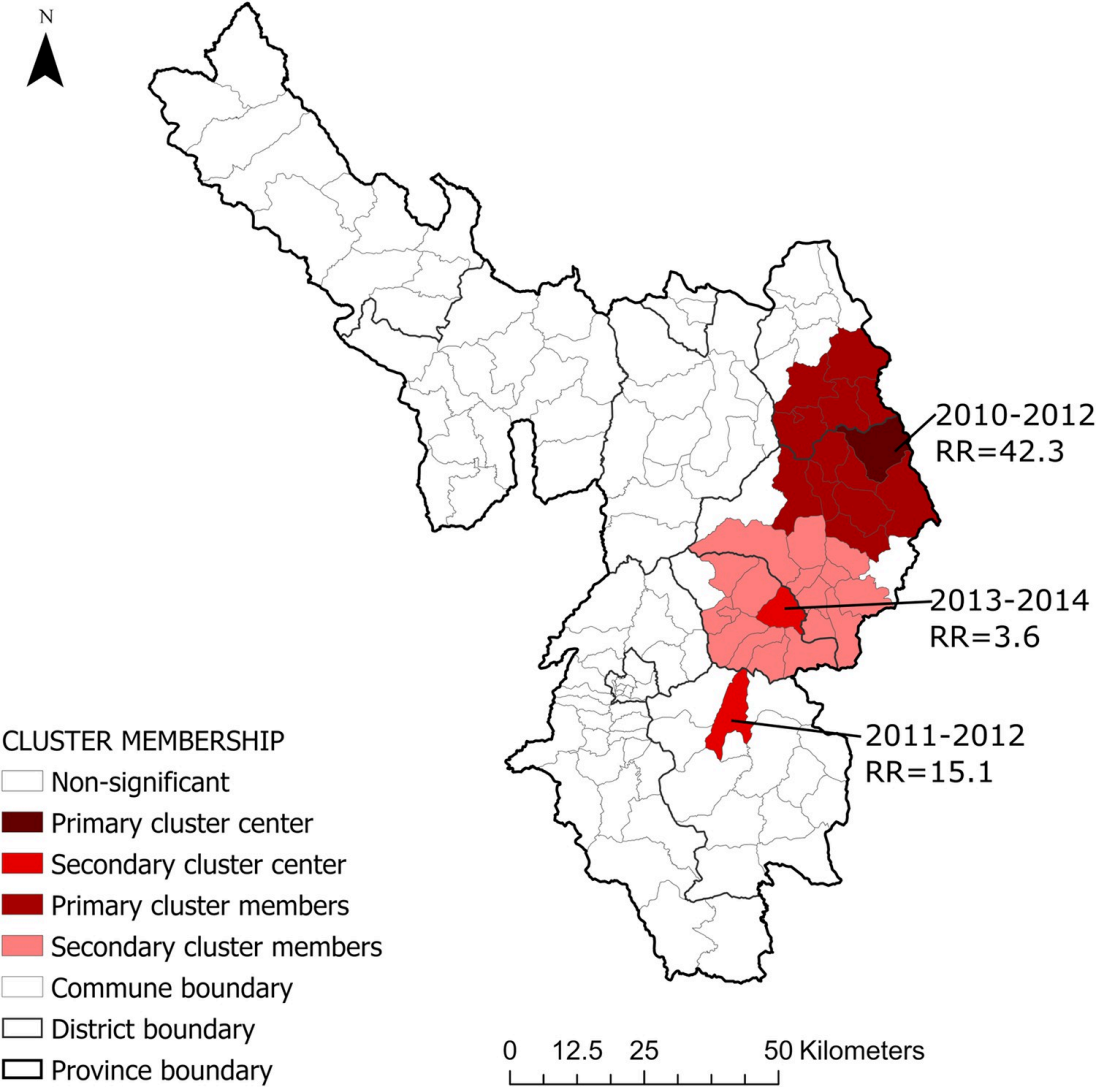
SaTScan-defined space-time clusters and relative risk of human anthrax (Poisson model, 999 permutations, 25% population at risk) in Dien Bien province, Vietnam. The space-time scan statistics were performed 15%, 25%, and 50% population at risk. Although the variation in the size of clusters were seen at different sizes of circles, the clusters persisted in the same areas in short periods, therefore, the results at 25% were reported (detail in S7 Fig. Maps produced in ArcGIS Pro using political boundary shapefiles from https://geodata.ucdavis.edu/gadm/gadm4.1/shp/gadm41_VNM_shp.zip.

Three space-time clusters were identified by space-time scan statistic (Poisson model, 999 permutations). The most likely cluster occurred between 2010 and 2012 in eastern part of the province. The relative risk of this cluster was 42.3, in other words, the communes inside the cluster were 42.3 times more likely to have cases of human anthrax than the communes outside of the cluster. There were two other clusters located in-between the east and southeast. Among those, one cluster was comprised of a single commune. This cluster occurred between 2011 and 2012 with a relative risk of 15.1. The third cluster was identified between 2013 and 2014, with a relative risk of 3.6 (Fig 3).

### Epidemiological characteristics of human and animal anthrax in Dien Bien (2010–2018)

During the study period, the Dien Bien PCDC reported 388 human anthrax cases with no mortalities, and the Dien Bien Sub-DAH reported 32 animal anthrax cases. All the human cases were reported as cutaneous anthrax (report of Dien Bien PCDC dated 02 November 2018).

**Fig 4:**
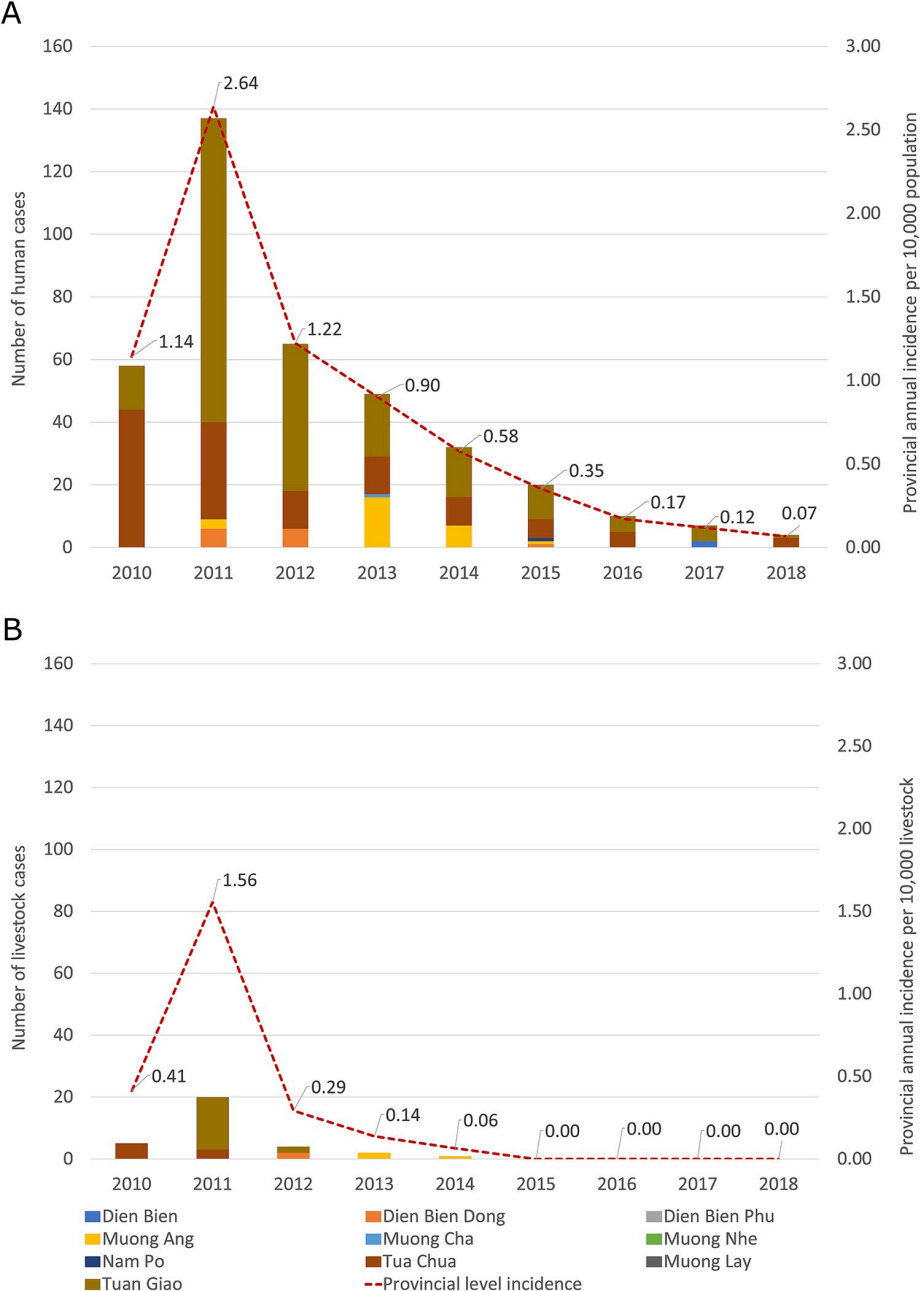
Annual trend of anthrax in human (A) and livestock (B) at provincial level and contribution of each district to the trends (2010–2018), Dien Bien province, Vietnam.

Fig 4 shows the annual incidence of human and animal anthrax at the provincial level (per 10,000) and number of cases at the district level. It indicates that the highest number of cases in humans and livestock was reported the first three years of study period with a peak in 2011 (nearly 140 cases in humans and 20 cases in animals). The annual incidence in human anthrax at the provincial level ranged between 0.1–2.64 per 10,000 human population with the upper value of the range reported in 2011. The incidence decreased dramatically after 2011 until 2018. A similar trend was seen for livestock with the range of annual incidence between 0–1.56 per 10,000 livestock. The highest animal incidence was also in 2011, then dropped remarkably to 0 from 2015 to 2018. Fig 4 illustrates the contribution of human and animal cases from 10 districts. Among those, Tuan Giao and Tua Chua remained the major sources of human cases throughout the period and for animal cases in 2010–2012. While Muong Ang and Dien Bien Dong emerged to contribute more cases in both humans and animals between 2012–2014. This suggests the co-occurrence of the disease in humans and animals but the occurrence of human cases while zero cases of animal anthrax reported between 2015–2018 requires more discussion. S8 Fig shows the annual disease trend at the district level.

**Fig 5:**
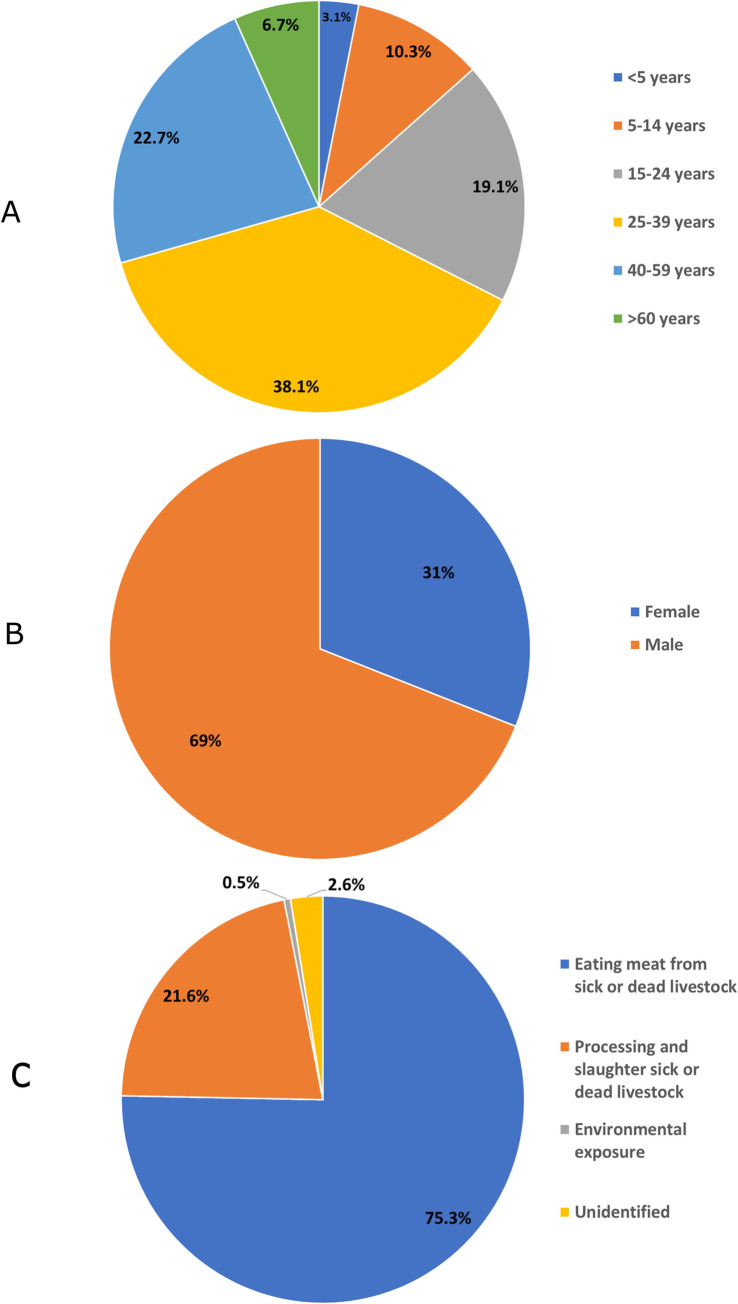
Epidemiological distribution of human anthrax reported in Dien Bien province Vietnam from 2012–2018 by age (A), gender (B) and source of infection (C).

Dien Bien PCDC had reported age, gender, and source of infection for 187 patients, which accounted for 48.9% of the total cases for the study period and 100% of those reported between 2012–2018 (187/187 cases) (Fig 5). The majority of cases were male (69%), aged between 15 and under 60 years old (79.9%), and 96.9% of these human cases had been involved in slaughtering, processing, or eating meat from sick or dead animals which are possible sources of infection. The remaining cases had unidentified sources or were suspected to be from environmental contamination.

**Fig 6:**
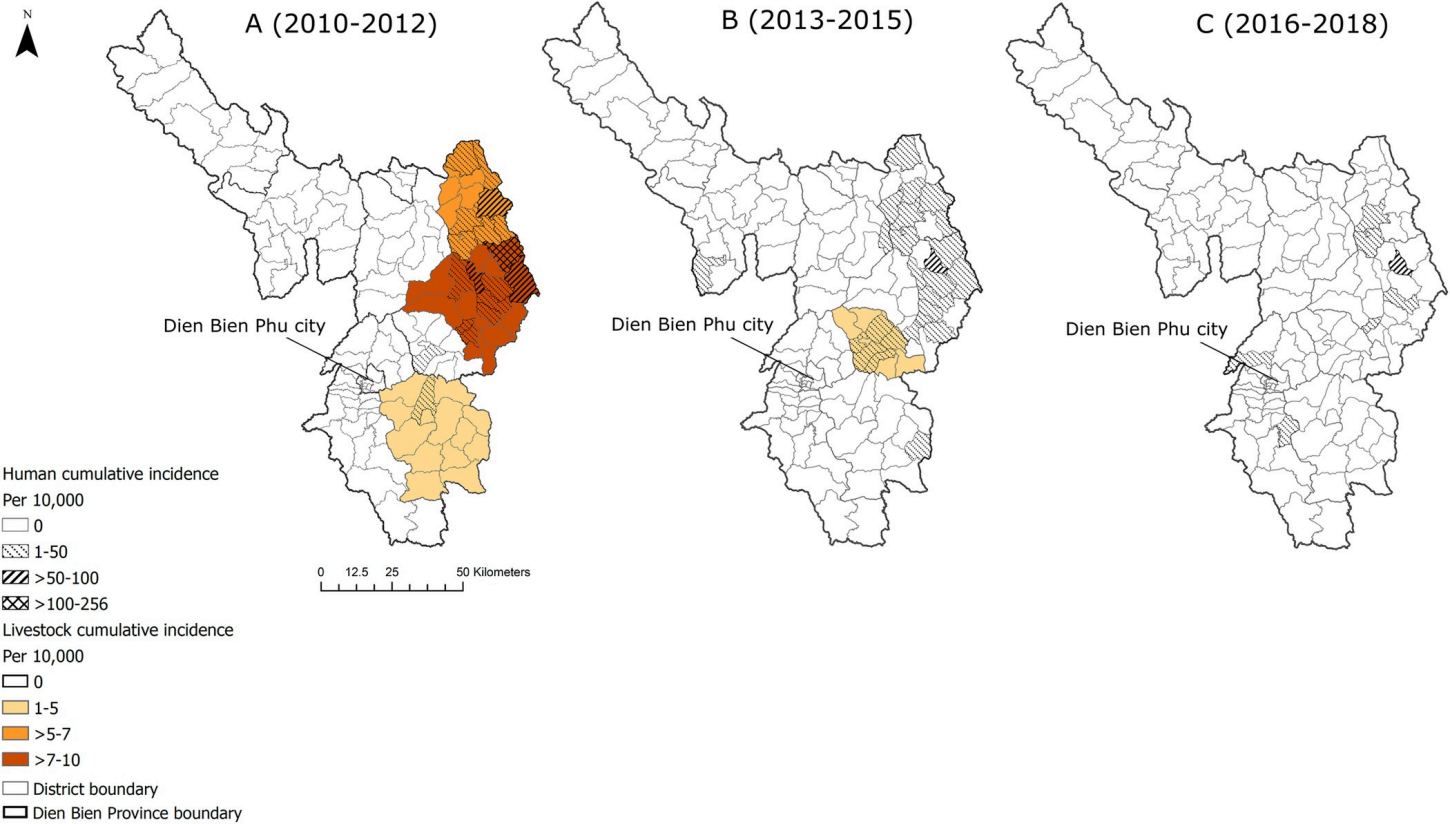
Choropleth maps identifying spatial overlap of human and livestock anthrax in 3-year intervals by crude cumulative incidence (per 10,000) for Dien Bien province, Vietnam. Maps produced in ArcGIS Pro using political boundary shapefiles from https://geodata.ucdavis.edu/gadm/gadm4.1/shp/gadm41_VNM_shp.zip.

Fig 6 indicates the overlap between human and animal anthrax in the east and southeast regions of Dien Bien province. The communes with higher incidence of human anthrax were seen in districts with higher incidence of anthrax in livestock (>7–10 cases per 10,000 livestock) (Fig 6A). However, anthrax in livestock was reported with very low incidence in some communes between 2013–2015 (Fig 6A), and zero cases were reported from 2016 to 2019 (Fig 6C), whereas human anthrax was present throughout the period.

### Incidence rates of anthrax in humans and livestock compared to annual livestock vaccine coverage

The annual incidence rates of anthrax in human and livestock from 2010 to 2019 in Dien Bien were compared to the annual coverage of anthrax vaccine in the livestock population (buffalos and domestic cattle) that the results are presented in S9 Fig. The estimated vaccine coverages are quite close to the annual data of 25% provided by Dien Bien Sub-DAH (report of Dien Bien PCDC and Sub-DAH, 2018).

**Fig 7:**
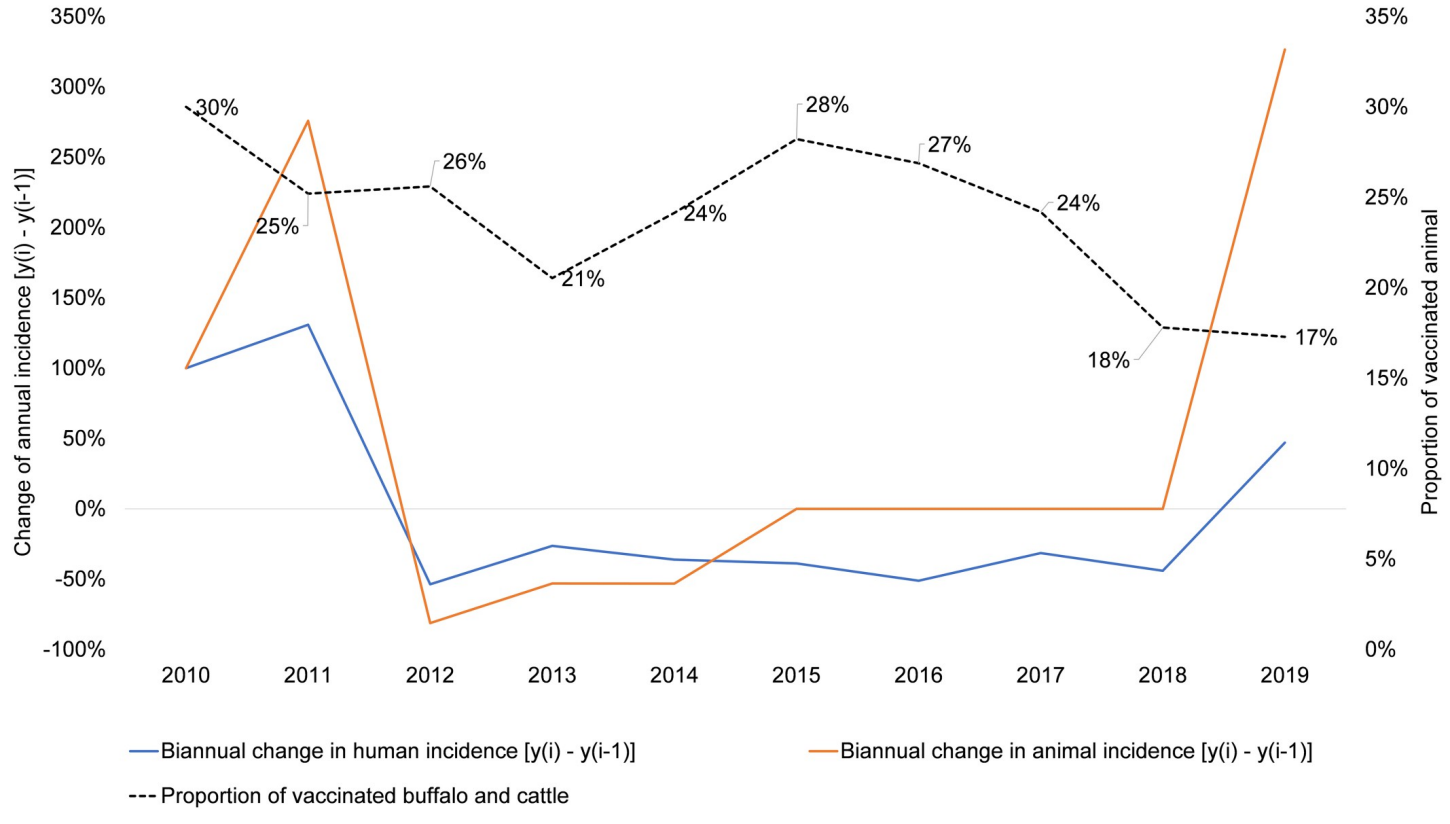
Association between vaccination coverage in livestock and biannual change in incidence of human and livestock anthrax (2010–2019), Dien Bien province, Vietnam.

Fig 7 plots annual livestock vaccine coverage and the inter-annual change in human or livestock incidence (2010 was used as reference year for itself (100%)). These results indicate a drop in vaccine coverage in a given year would associate with an incidence increase of both human and animal anthrax in the year after (e.g., 2011, 2013, 2017, 2019), with some years showing a dramatic increase. There were no reported livestock cases from 2015 to 2018, so the graphed increase of livestock anthrax in 2019 was the comparison of 2019 to 2014.

## Discussion

This study examined the spatial patterns and epidemiological characteristics of human and livestock anthrax, and livestock anthrax vaccination efforts from 2010–2019 in Dien Bien Province, Vietnam. Our results indicate localized anthrax case persistence in humans and livestock despite livestock vaccine control efforts.

Localized persistent of human anthrax within the province was identified throughout the study period while zero to very low incidence of livestock anthrax was reported in the second half of the study period. It is an interesting pattern since human anthrax is strongly associated with anthrax in animals. For example, Bangladesh and Kazakhstan showed all reported human cases had historical contact with animal carcasses or were involved in slaughtering and/or processing infected meat [3,4]. Other studies indicated dramatically increased risk of human anthrax among people involved in handling, butchering, skinning, cutting meat, eating meat of animals suspected to have anthrax [2,3]. Here, data on the source of infection from Dien Bien identified 96.9% of cases were exposed to anthrax by eating or participating in slaughtering sick or dead livestock. Data from Dien Bien were anthropocentric since the anthrax surveillance had been mainly based on the notification of human anthrax cases by the human health system in the province. This anthropocentrism of anthrax reporting has been noted elsewhere [30].

The absence of livestock anthrax cases in the second half of the study period could be explained by two possible hypotheses. First, as anthrax is neglected in many regions of the world [31], it could be highly underreported for livestock in Dien Bien province. This is particularly true when livestock is a valuable household asset and the value of the meat could be lost if reported [13]. Toward this, the Vietnamese Law of Infectious Disease and Control in 2007 mentioned compensation for financial losses of livestock owners in order to encourage them to notify local authorities about livestock dying of certain diseases [32]. However, limited community awareness about the regulation, as well as limited knowledge of livestock clinical signs, could be limiting the communities’ levels of reporting. Such gaps were identified in an assessment of joint Circular 16 which is arguably the most important regulatory document for One Health surveillance and interventions in Vietnam and identifies anthrax as one of the prioritized diseases [33]. The assessment conducted by Food and Agriculture Organization (FAO) in 2016 indicated gaps in awareness of human and animal health staff about the circular at local levels, such as communes and districts (our spatial units of analysis here) [16].

According to the Dien Bien PCDC in 2018, there are many challenges for anthrax surveillance in the province. These include difficulty in transportation to remote areas of the province where the communication system remains less developed. There are also constraints in language, customs, and cultural practices among ethnic minorities whose education level limits access to information on disease prevention measures. Transportation difficulties lead to late notification of anthrax cases, delayed sample collection, and often result in failure of laboratory diagnostic confirmation when antibiotics were already administered [34]. This latter point is particularly important in Vietnam, where antibiotics are widely available and often without prescription, though national laws require them [35]. Furthermore, laboratory capacity is important for sample quality assurance, which is insufficient locally for timely sample collection and preservation. Financial and human resources for disease surveillance and response are lacking in Dien Bien province, especially for the animal health sector when veterinary institutions have been merged into the husbandry and agricultural services at provincial and district levels. No official veterinary institution is available at the commune level in the province. This system reform could result in a shortage of competent animal health staff for surveillance and response in the long run, particularly in the communes and district.

A second explanation for the mismatch between the number of human cases and livestock cases during the second half of the study period could be that the source of infection for human anthrax in the province was not only because of close contact between humans and livestock. Although it is scarce, other source of infection could be direct exposure of human to spores of *Bacillus anthracis* persisted in environment which could be a reasonable explanation for the infection among children under 5 years as discussed in following section [36,37]. Another source of infection might involve necrophagous flies as a vector for transmission of anthrax from unreported animal carcasses (wildlife or domestic livestock) to humans living nearby the carcasses’ location through food contamination [36,38]. Flies could play a role in anthrax transmission and should be further studied. Although there is no direct evidence on the transmission of anthrax via necrophagous flies in Vietnam, the literature supports that there are species of necrophagous flies (family *Sarcophagidae*) recorded in Vietnam [39], which have been confirmed to carry and deposit *Bacillus anthracis* in studies in Texas in the USA [38,40].

The epidemiological characteristics of human anthrax suggest that there are patterns in the types of anthrax infection, patient gender, age, and source of infection. In our study, all patients with available information were diagnosed with cutaneous anthrax; 96.9% had a history of exposure from slaughtering and processing meat from sick or dead livestock. Previous studies in Bangladesh and Kazakhstan provide similar results that cutaneous anthrax was the predominant type among patients [3,4]. Another study in Uganda on a gastrointestinal anthrax outbreak showed that 82% of the patients had a history of eating meat from dead animals that had unknown causes of death [41]. The findings of our study warrant further investigation to reveal the practices used to process and/or consume meat of sick or dead animals since there was no gastrointestinal anthrax reported in the study period. Regarding gender, 69% of patients were male, which is higher than studies in Bangladesh and Uganda (57.4% and 47.5%, respectively) [4,41], but lower than in a study in Republic of Georgia (84%) [30]. In Dien Bien, 60.8% patients aged between 25–59 years old, though remarkably high percentages of human anthrax occurred in lower age groups (19.1% among 15–24 years old; 10.3% among 5–14 years old; and 3.1% under 5 years of age). These findings are quite similar to the study in Uganda [41] and could be explained by meat sharing practices among households in the mountainous areas of Vietnam where the livestock owners gather relatives and neighbors to slaughter and eat together, however it does not address the occurrence of anthrax in small children under 5. Another explanation is that the children under 15 years of age were involved in livestock grazing with frequent exposure to livestock and bacterium spores in grazing areas. A report of International Labour Organization (ILO) in 2012 indicated that one-sixth of Vietnamese children under 17 years old, most likely to be male, living in rural areas, were involved in some forms of economic activity, and 70% were involved in agricultural works like cultivation and animal husbandry [42], but this connection needs to be explored further.

The spatial distribution of human and animal anthrax was overlapping in this study, which were in eastern districts (livestock) and communes (human) of the province. Space-time clustering analysis indicated the clusters of human anthrax occurred from 2010 to 2014 with the most likely space-time cluster from 2010 to 2012. However, local Moran’s I statistics revealed clusters of human anthrax persisted in the area throughout study period. Human cases were more sporadic from 2015 to 2018 compared to the large outbreaks earlier in the study period (2010–2014). This pattern could be due to the implementation of a livestock anthrax vaccine campaign during the study period with a peak of 28% bovid (cattle, buffalo) coverage in 2015 (S9 Fig. A long-term study of anthrax in Azerbaijan revealed a significant decrease in human anthrax in Azerbaijan following the re-establishment of a national livestock vaccination program after Soviet independence [43]. In contrast, a study in neighboring Georgia revealed the opposite, with a drastic increase in human anthrax after the national compulsory vaccination policy ended [44]. Our data, like those previous studies, highlight the importance of livestock vaccination to reduce human anthrax incidence. The drastic increase of both human and livestock anthrax cases in 2019 in Dien Bien could serve as a warning for new outbreaks as vaccination coverage has been well below 28%. Notably, the increase in cases in Georgia was biased toward ethnic minority groups (Azerbaijani and Armenian). In Vietnam, ethnic minority groups are highly involved in agriculture and livestock handling and may see similar disproportional risks in Dien Bien. Anthrax is known to be a periodic disease [36,45], so we cannot rule out some of the annual increases in Dien Bien were partially due to conditions promoting larger outbreaks but the increase in cases in years following vaccine declines was seen throughout our study period.

The current study identified spatial clusters of high anthrax risk and increasing incidence in humans and livestock each time livestock vaccine coverage dropped in Dien Bien province. However, there are still limitations of available data on human and animal anthrax, which might be applicable to other provinces of Vietnam. First, data on community awareness on anthrax and cultural practices were not available to explain the distribution and persistence of anthrax in the province. Second, the mismatch of human and animal cases cannot be fully explained when the number of human cases were higher than the number of animal cases. Work is needed to determine if anthrax in livestock is underreported and/or if individual animal carcasses are responsible for very high numbers of human cases through meat sharing.

## Conclusion

The current study identified the spatio-temporal distribution and persistence of human and livestock anthrax, which may allow Dien Bien province to better target surveillance and livestock vaccination campaigns. Our work suggests a clear pattern in increased human cases when vaccination efforts wain, helping to better target long term vaccination in hotspots defined here. While differences in sociocultural practices and disease surveillance may limit the value of these results in other provinces, these analyses provide a framework for using spatio-temporal analyses to define risk areas for anthrax persistence in northern Vietnam applicable beyond Dien Bien province. Further work is needed to address the data limitations and provide more explanation on the disease distribution regarding community awareness and cultural practices associated with anthrax in humans and animals, and the situation of animal anthrax surveillance in the province.

## Supporting information

S1 Text Case definitions for anthrax in humans and livestock in Vietnam. (DOCX)

S1 Fig Provincial, District, and commune level administration of Dien Bien province. Maps produced in ArcGIS Pro using political boundary shapefiles from https://geodata.ucdavis.edu/gadm/gadm4.1/shp/gadm41_VNM_shp.zip.
(TIFF)

S2 Fig Comparison of human population (by National census in 2009, 2019 and annual growth rate) and livestock population (provided by Dien Bien Sub-DAH) at provincial level versus estimation by Zonal statistics tool for human (A) and livestock (B).
(TIFF)

S3 Fig Comparison between crude, Empirical Bayes Smoothed, and Spatial Bayes Smoothed cumulative incidence of human anthrax in 3-year intervals (A: 2010–2012; B: 2013–2015; C: 2016–2018).
(TIFF)

S4 Fig **Comparing distribution of human anthrax by crude and Spatial Bayes Smoothed cumulative incidence (per 10,000) at commune level in Dien Bien province in every 3-year intervals (A, B, C for crude CI, and D, E, F for SBS CI).** Maps produced in ArcGIS Pro using political boundary shapefiles from https://geodata.ucdavis.edu/gadm/gadm4.1/shp/gadm41_VNM_shp.zip.
(TIFF)

S5 Fig **Population increases at district level between 2010–2018 for human (A) and livestock (D); and Population distribution in each 2010 or 2018 for human (B, C) and livestock (E, F) by Zonal statistics (human, livestock) and growth rates (livestock only).** Maps produced in ArcGIS Pro using political boundary shapefiles from https://geodata.ucdavis.edu/gadm/gadm4.1/shp/gadm41_VNM_shp.zip.
(TIFF)

S6 Fig Results of Local Moran’s I statistics for Spatial Empirical Based Smoothed cumulative incidence of human anthrax in 3-year intervals (A: 2010–2012; B: 2013–2015; C: 2016–2018).
(TIFF)

S7 Fig SaTScan statistics defined Space-time clusters and relative risk of communes inside over outside of clusters (Poisson model, 999 permutations, 15%, 25% and 50% population at risk). Maps produced in ArcGIS Pro using political boundary shapefiles from https://geodata.ucdavis.edu/gadm/gadm4.1/shp/gadm41_VNM_shp.zip.
(TIFF)

S8 Fig Annual trend of human anthrax (A) and livestock anthrax (B) at provincial and district levels (2010–2018).
(TIFF)

S9 Fig Association between anthrax vaccination coverage in livestock and the incidence of anthrax in human and livestock (2010–2019). (TIF)
