# Supplementary material for: Spatial analysis of human and livestock anthrax in Dien Bien province, Vietnam (2010–2019) and the significance of anthrax vaccination in livestock
Source: PLoS Negl Trop Dis. 2022 Dec 20;16(12):e0010942. doi: 10.1371/journal.pntd.0010942 (PMC9767330; doi:10.1371/journal.pntd.0010942)
Supplement: S1 Text — (DOCX) [file pntd.0010942.s001.docx]

**SUPPLEMENT**

Spatial analysis of human and livestock anthrax in Dien Bien province, Vietnam (2010-2019) and the significance of anthrax vaccination in livestock

Luong Minh Tan^1,2,5‡^, Doan Ngoc Hung^3‡^, Do Thai My^4^, Morgan A. Walker^1,2^, Hoang Thi Thu Ha^5^, Pham Quang Thai^5,6^, Tran Thi Mai Hung^5^, Jason K. Blackburn^1,2*^

1. **Clinical signs and symptoms for surveillance of anthrax in human and livestock**

**Human anthrax case definition (Decision number 5703/QD-BYT):**

- Suspected/clinical case is a person who exposes to animal, animal product that is suspected of animal anthrax or living in endemic area. The acute onset signs and symptoms fall into one of the following categories:
  - Cutaneous anthrax: itchy in the infection site, then it forms small blisters, painless black eschar, normally seen around arm, hand, around the patient’s mouth and knee.
  - Inhalation anthrax: pneumonia-like symptoms but rapid progress to hard breathing and septic shock.
  - Gastrointestinal anthrax: remarkable abdominal pain, fever, and septic shock.
  - Meningitis anthrax: acute onset, seizure, lost consciousness, and other signs and symptoms of meningitis infection.
- Confirmed case is a suspected/clinical case with one of the following confirmatory tests: bacterium identification by culture, or typical genetic material by molecular biological techniques (PCR).

**Livestock anthrax case definition (Circular number 07/2016/TT-BNNPTNT):**

The signs of anthrax in livestock include tongue exposure, abdominal distention, body fluid containing dark and non-clotting blood run out from mouth, nose, anus, and genital parts. Other signs and symptoms are fever (40^o^C-42.5^o^C), high heartbeat rate, shortened breathing diarrhea, reduced milk production, abortion, staggering walk, seizure, red eyes. In some cases, the livestock bumps into a bush and die suddenly or die after 1-3 days of the onset. Cutaneous symptoms are swollen areas in neck, chest, rump that become cold later, painless, rotten eschar, sometimes forming dark red eschar with yellow fluid. Incubation period in 3-7 days with some exception of 2 days or up to 2 weeks.

**Supplement figure headings**

**S1 Fig. Provincial, District, and commune level administration of Dien Bien province.** Maps produced in ArcGIS Pro using political boundary shapefiles from https://geodata.ucdavis.edu/gadm/gadm4.1/shp/gadm41_VNM_shp.zip.

**S2 Fig. Comparison of human population (by National census in 2009, 2019 and annual growth rate) and livestock population (provided by Dien Bien Sub-DAH) at provincial level versus estimation by Zonal statistics tool for human (A) and livestock (B).**

**S3 Fig. Comparison between crude, Empirical Bayes Smoothed, and Spatial Bayes Smoothed cumulative incidence of human anthrax in 3-year intervals (A: 2010-2012; B: 2013-2015; C: 2016-2018).**

**S4 Fig. Comparing distribution of human anthrax by crude and Spatial Bayes Smoothed cumulative incidence (per 10,000) at commune level in Dien Bien province in every 3-year intervals (A, B, C for crude CI, and D, E, F for SBS CI).** Maps produced in ArcGIS Pro using political boundary shapefiles from https://geodata.ucdavis.edu/gadm/gadm4.1/shp/gadm41_VNM_shp.zip.

**S5 Fig. Population increases at district level between 2010-2018 for human (A) and livestock (D); and Population distribution in each 2010 or 2018 for human (B, C) and livestock (E, F) by Zonal statistics (human, livestock) and growth rates (livestock only).** Maps produced in ArcGIS Pro using political boundary shapefiles from https://geodata.ucdavis.edu/gadm/gadm4.1/shp/gadm41_VNM_shp.zip.

**S6 Fig. Results of Local Moran’s I statistics for Spatial Empirical Based Smoothed cumulative incidence of human anthrax in 3-year intervals (A: 2010-2012; B: 2013-2015; C: 2016-2018).**

**S7 Fig. SaTScan statistics defined Space-time clusters and relative risk of communes inside over outside of clusters (Poisson model, 999 permutations, 15%, 25% and 50% population at risk).** Maps produced in ArcGIS Pro using political boundary shapefiles from https://geodata.ucdavis.edu/gadm/gadm4.1/shp/gadm41_VNM_shp.zip.

**S8 Fig. Annual trend of human anthrax (A) and livestock anthrax (B) at provincial and district levels (2010-2018).**

**S9 Fig. Association between anthrax vaccination coverage in livestock and the incidence of anthrax in human and livestock (2010-2019).**
