# Supplementary figures and images for: Spatial analysis of human and livestock anthrax in Dien Bien province, Vietnam (2010–2019) and the significance of anthrax vaccination in livestock
Source: PLoS Negl Trop Dis. 2022 Dec 20;16(12):e0010942. doi: 10.1371/journal.pntd.0010942 (PMC9767330; doi:10.1371/journal.pntd.0010942)

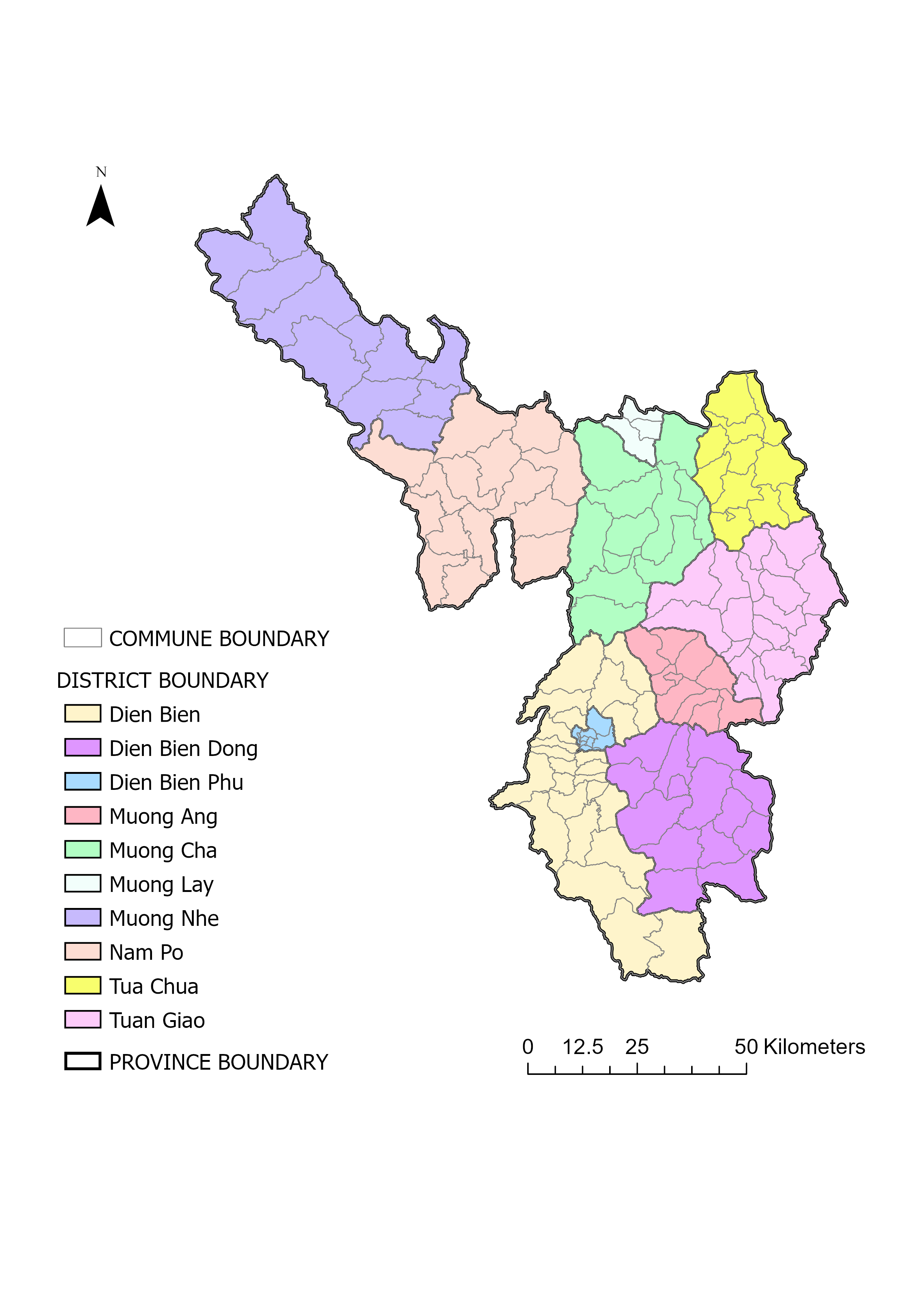

Supplement: S1 Fig — Maps produced in ArcGIS Pro using political boundary shapefiles from https://geodata.ucdavis.edu/gadm/gadm4.1/shp/gadm41_VNM_shp.zip. (TIFF) [file pntd.0010942.s002.tiff]

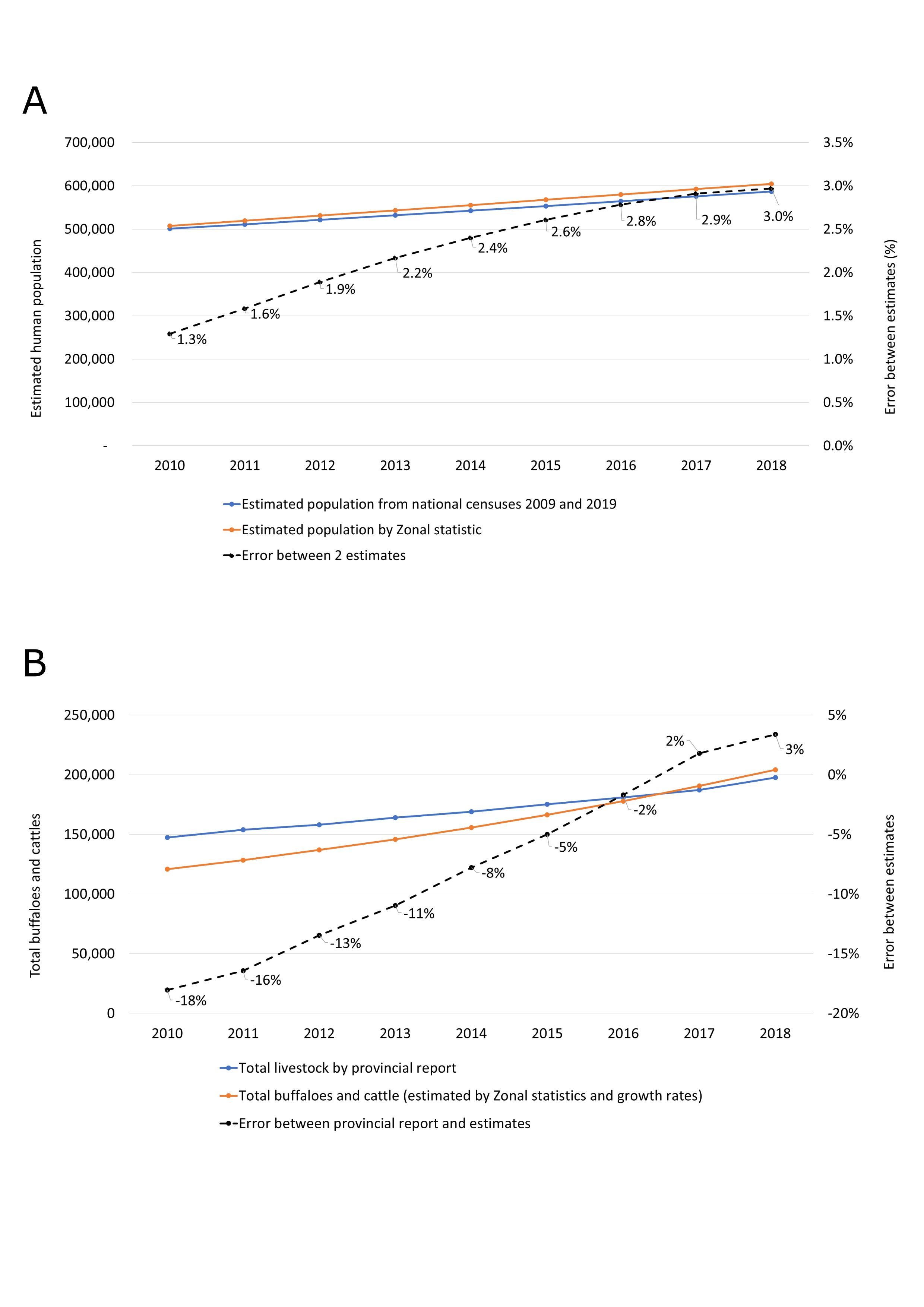

Supplement: S2 Fig — Comparison of human population (by National census in 2009, 2019 and annual growth rate) and livestock population (provided by Dien Bien Sub-DAH) at provincial level versus estimation by Zonal statistics tool for human (A) and livestock (B). (TIFF) [file pntd.0010942.s003.tiff]

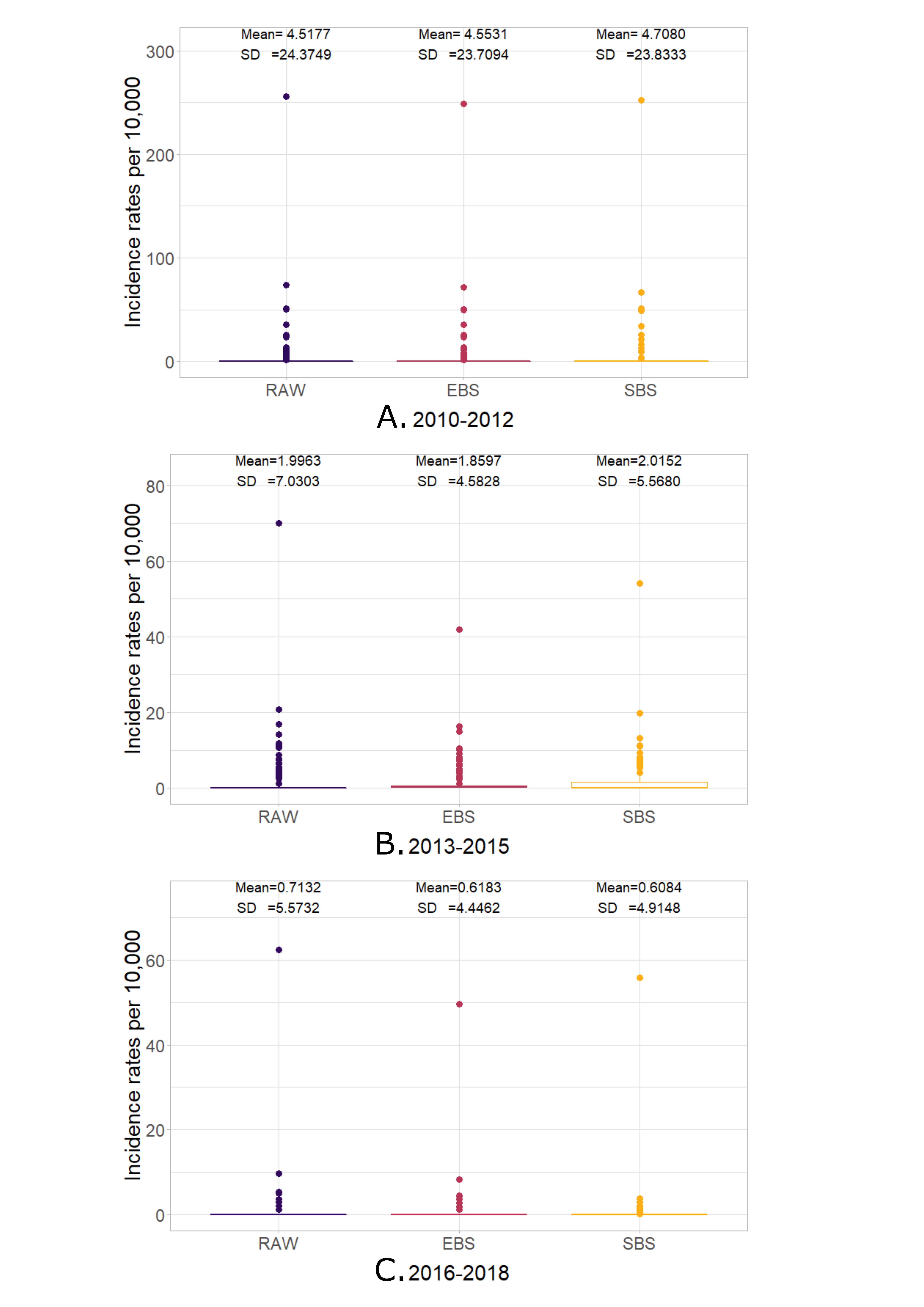

Supplement: S3 Fig — Comparison between crude, Empirical Bayes Smoothed, and Spatial Bayes Smoothed cumulative incidence of human anthrax in 3-year intervals (A: 2010–2012; B: 2013–2015; C: 2016–2018). (TIFF) [file pntd.0010942.s004.tiff]

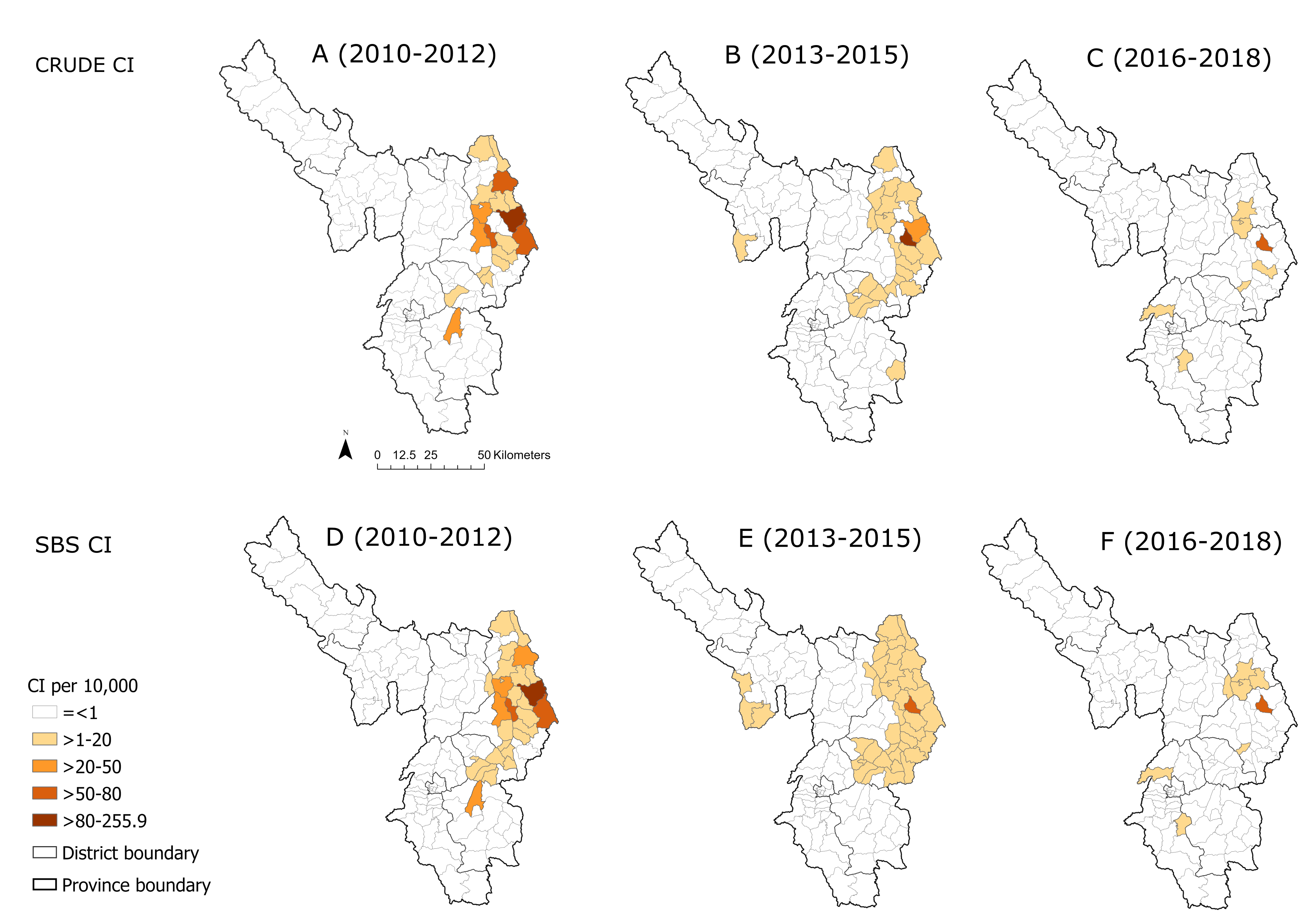

Supplement: S4 Fig — Comparing distribution of human anthrax by crude and Spatial Bayes Smoothed cumulative incidence (per 10,000) at commune level in Dien Bien province in every 3-year intervals (A, B, C for crude CI, and D, E, F for SBS CI). Maps produced in ArcGIS Pro using political boundary shapefiles from https://geodata.ucdavis.edu/gadm/gadm4.1/shp/gadm41_VNM_shp.zip. (TIFF) [file pntd.0010942.s005.tiff]

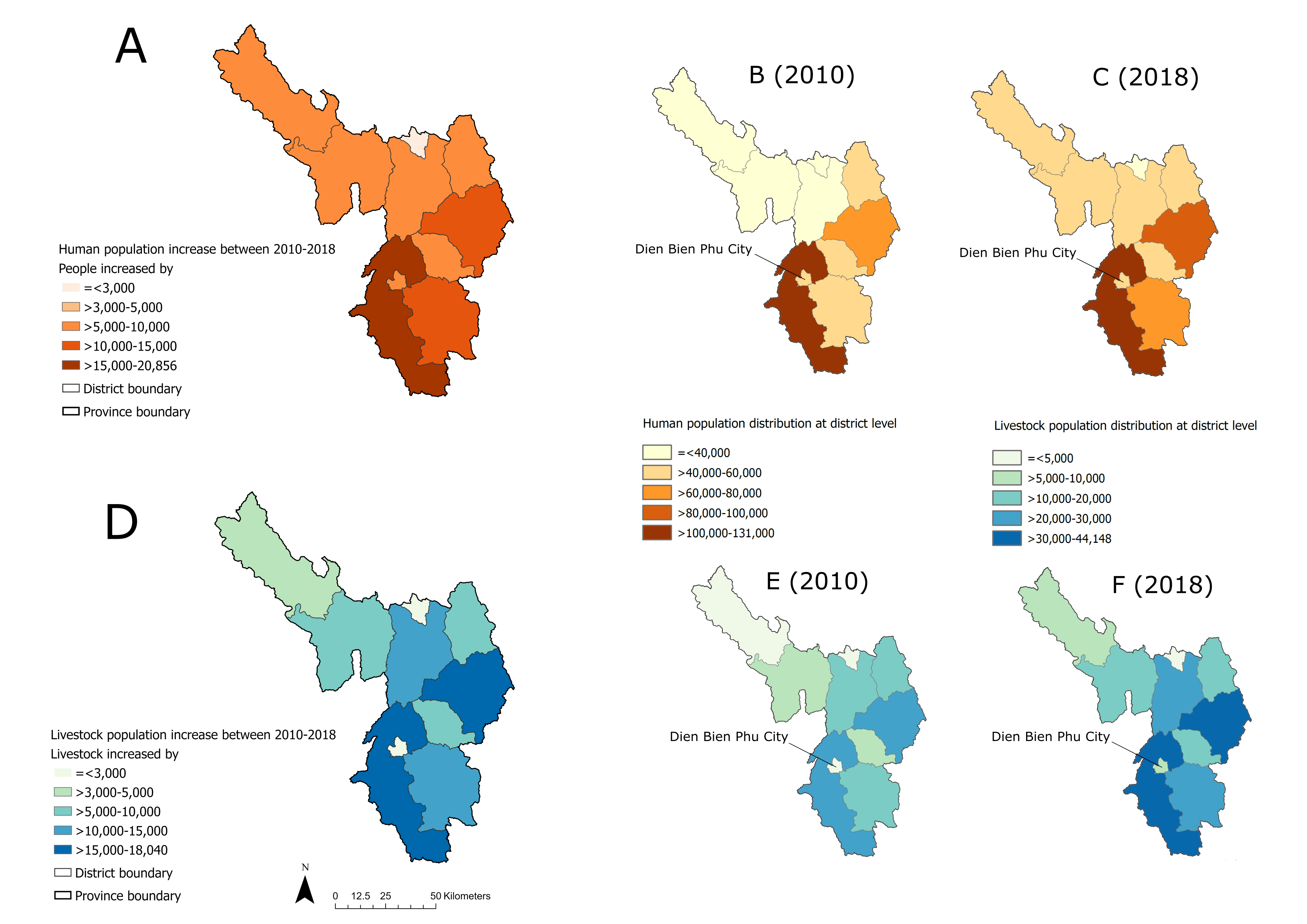

Supplement: S5 Fig — Population increases at district level between 2010–2018 for human (A) and livestock (D); and Population distribution in each 2010 or 2018 for human (B, C) and livestock (E, F) by Zonal statistics (human, livestock) and growth rates (livestock only). Maps produced in ArcGIS Pro using political boundary shapefiles from https://geodata.ucdavis.edu/gadm/gadm4.1/shp/gadm41_VNM_shp.zip. (TIFF) [file pntd.0010942.s006.tiff]

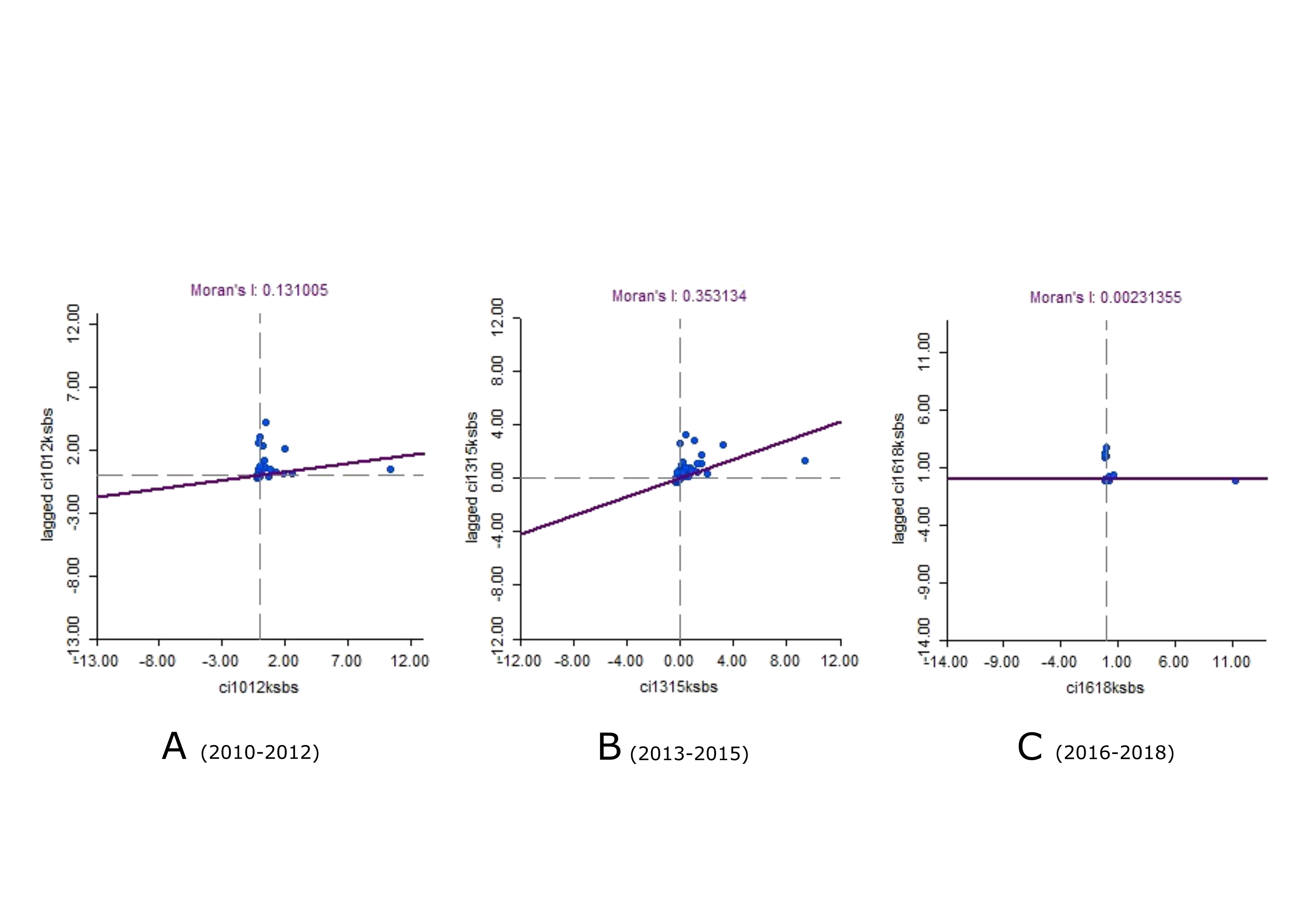

Supplement: S6 Fig — Results of Local Moran’s I statistics for Spatial Empirical Based Smoothed cumulative incidence of human anthrax in 3-year intervals (A: 2010–2012; B: 2013–2015; C: 2016–2018). (TIFF) [file pntd.0010942.s007.tiff]

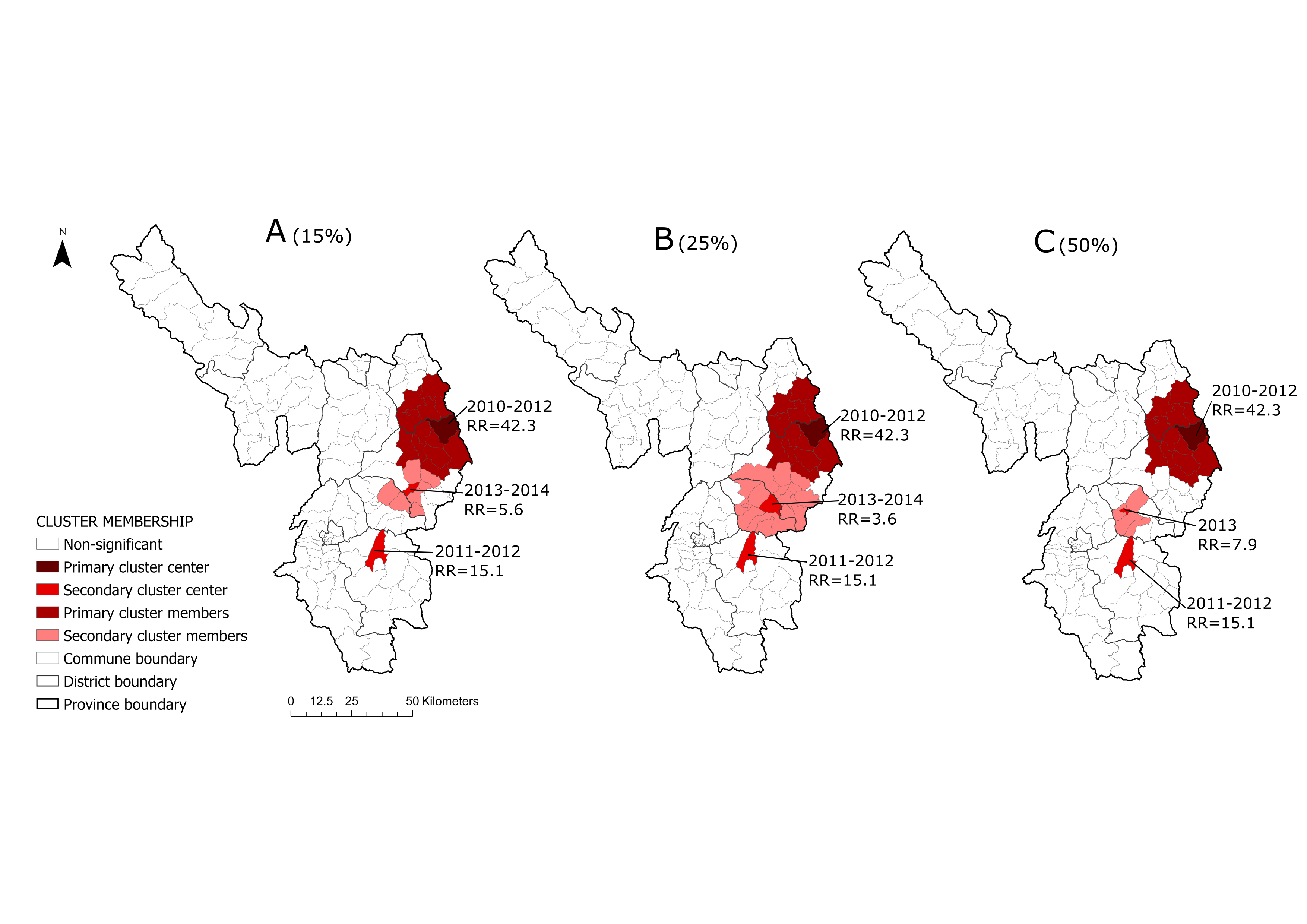

Supplement: S7 Fig — Maps produced in ArcGIS Pro using political boundary shapefiles from https://geodata.ucdavis.edu/gadm/gadm4.1/shp/gadm41_VNM_shp.zip. (TIFF) [file pntd.0010942.s008.tiff]

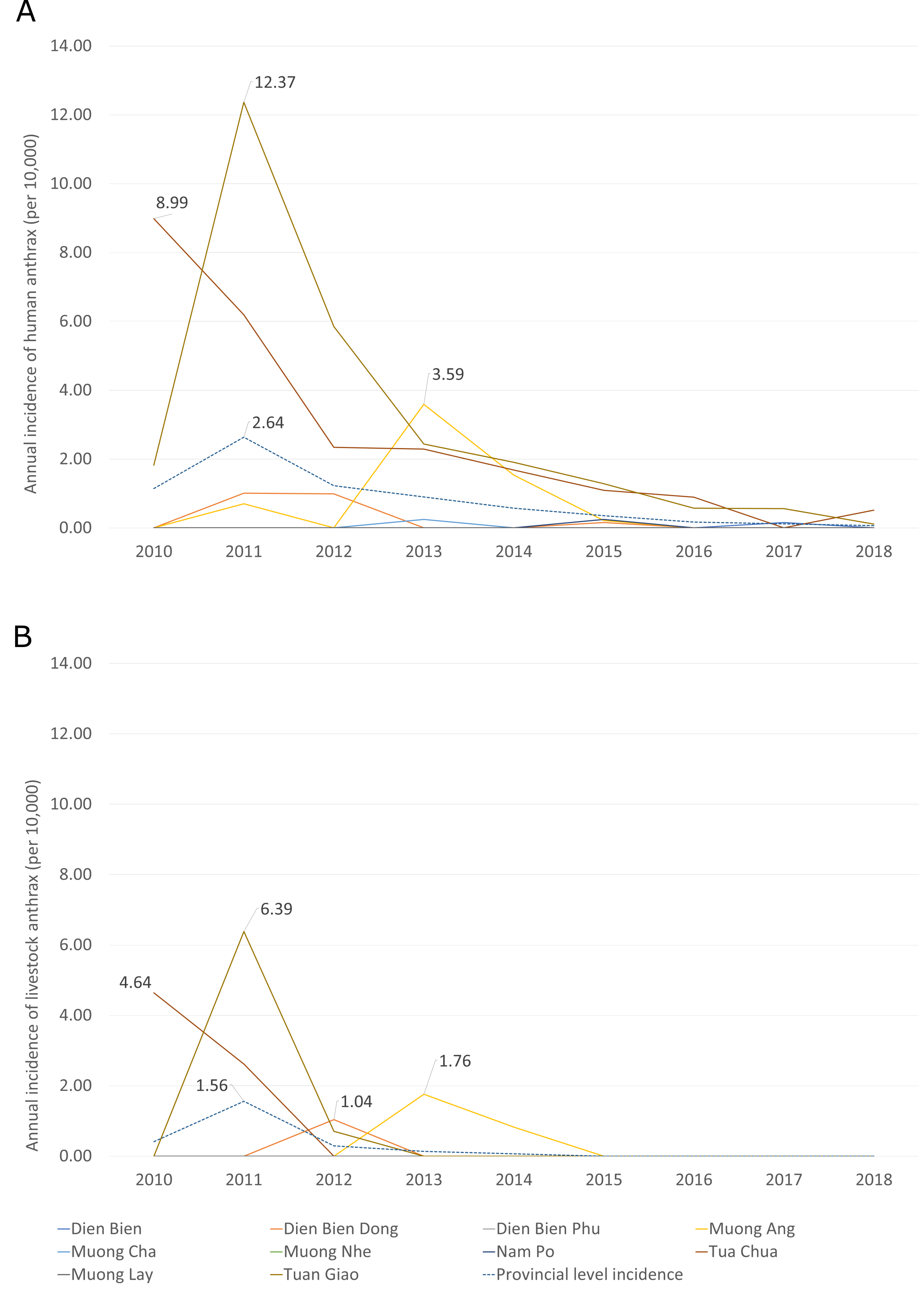

Supplement: S8 Fig — Annual trend of human anthrax (A) and livestock anthrax (B) at provincial and district levels (2010–2018). (TIFF) [file pntd.0010942.s009.tiff]

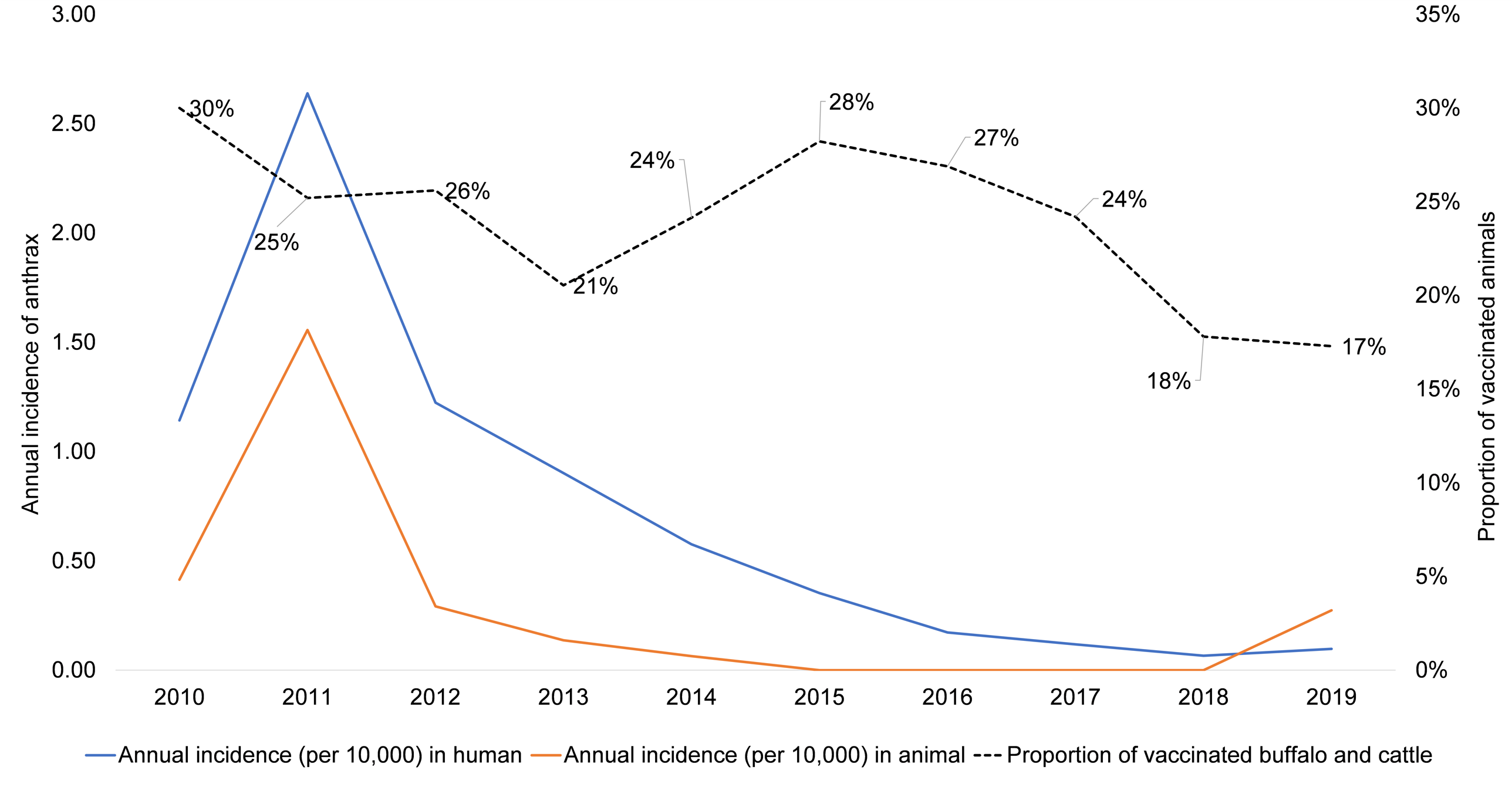

Supplement: S9 Fig — (TIF) [file pntd.0010942.s010.tif]
